# Discovery of novel small molecule Asparaginyl endopeptidase inhibitors via dual approach‐based virtual screening and molecular simulation studies

**DOI:** 10.1002/alz.093291

**Published:** 2025-01-09

**Authors:** MEENAKSHI SINGH, Joyal Xavier, Sampada P Tamhankar, Raj Amin

**Affiliations:** ^1^ Auburn University, AUBURN, AL USA

## Abstract

**Background:**

Neurological disorders are at epidemic levels in the world today. Various proteins are being targeted for the development of novel molecular therapeutics; however, no small‐molecule inhibitors have been discovered. Recent studies suggest that there are few molecules in clinical trials for various secretase (α, β, and γ), caspase, and calpain inhibitors. However, recently an emerging target was highlighted i.e. asparaginyl endopeptidase (AEP). This lysosomal cysteine protease cleaves the glycine‐rich C‐terminal to form an asparagine residue. In addition, the highly conserved and spatially close catalytic triad (Oδ1_ASN42_‐Nε2_HIS148_ ~ 3.0Å) active site residue is Asn 42, His 148‐spacer‐Cys 189, which is responsible for proteolytic activity are also impacted by the cleavage of the C‐terminal region of AEP. Therefore, the current study focuses on screening, available small molecule databases for potential interactions with the active binding sites within the AEP binding pocket for inhibiting its proteolytic activity and restoring neuronal damage.

**Method:**

Various online available CNS‐focused databases were screened to find small molecule inhibitors using an *in‐silico* structure‐based virtual screening method and molecular dynamics simulation. Further selected small molecules were assayed on overexpressed AEP stable cell line (HMC‐3) with exiting peptide and compound 11

**Result:**

There is no small molecule inhibitor for the AEP yet, and the screened small molecule could be a potential hit for drug discovery. In addition, we have observed that AEP is involved in various proteinopathies which leads to neuroinflammation and can be reversed. Moreover, we found that our *in‐silico* screened molecules are more potent than the compound 11 and peptide. Further assays on a stable AEP immortal HMC‐3 cell line also line up with the *in‐silico* results.

**Conclusion:**

The selected small molecule presented a potential non‐covalent interaction (hydrogen bonds, ionic bonds, and pi‐pi interaction) within the catalytic binding sites with high binding energies and stable complex during simulation when compared to exiting compound 11 and peptide. The findings from the current study will help identify the critical subgroups needed for the development of our lead AEP inhibitory molecule.

